# Serum 8-iso-PGF2α Predicts the Severity and Prognosis in Patients With Community-Acquired Pneumonia: A Retrospective Cohort Study

**DOI:** 10.3389/fmed.2021.633442

**Published:** 2021-03-29

**Authors:** Ling Zheng, Jun Fei, Chun-Mei Feng, Zheng Xu, Lin Fu, Hui Zhao

**Affiliations:** ^1^Respiratory and Critical Care Medicine, Second Affiliated Hospital of Anhui Medical University, Hefei, China; ^2^Department of Toxicology of Anhui Medical University, Hefei, China

**Keywords:** CAP, 8-iso-PGF2α, inflammatory cytokines, CAP severity scores, biomarker

## Abstract

**Background:** Many studies have identified the important role of 8-isoprostane (8-iso-PGF2α) in pulmonary diseases. However, the role of 8-iso-PGF2α in community-acquired pneumonia (CAP) remains unclear. Therefore, the main goal was to investigate the correlations of serum 8-iso-PGF2α with the severity and prognosis in CAP patients through a hospital-based retrospective cohort study.

**Methods:** All 220 patients with CAP were enrolled. Demographic information and clinical data were collected. Levels of 8-iso-PGF2α and inflammatory cytokines were detected in serum using ELISA.

**Results:** The levels of 8-iso-PGF2α were gradually increased in parallel with the CAP severity scores. Univariate and multivariate logistic regression analyses revealed a positive association between serum 8-iso-PGF2α and the CAP severity scores. Additionally, serum 8-iso-PGF2α levels were positively correlated with circulating inflammatory cytokines (CRP and TNFα). Serum 8-iso-PGF2α levels were increased in the patients with a longer hospital stay than those with a shorter hospital stay. Additionally, 20 patients died after hospitalization. Dead patients presented a higher serum 8-iso-PGF2α than surviving patients. A subsequent survival analysis revealed that higher serum 8-iso-PGF2α levels positively correlated with the risk of death in patients with CAP.

**Conclusions:** Serum 8-iso-PGF2α levels on admission are positively associated with the severity of CAP patients. Elevated serum 8-iso-PGF2α on admission prolongs hospital stay and increases the risk of death in patients with CAP, indicating that 8-iso-PGF2α may be involved in the progression of CAP and serve as an early serum prognostic biomarker for CAP.

## Introduction

Community-acquired pneumonia (CAP) is an infectious disease caused by bacteria, viruses, or several mixed infections. CAP has gradually become a significant cause of morbidity and mortality despite advancements in antibiotic treatment all over the world (1–3). CAP has a high prevalence in patients receiving treatment in the intensive-care unit. The incidence in adults is 1.6 to 11 per 1,000 adults in different countries, with rates of hospitalization between 40 and 60%. There are more than 4 million adults, accounting for more than 1 million hospital admissions per year in America. CAP has become the sixth leading cause of death (4, 5). More seriously, pneumonia is the most primary cause of death in children (6). Immediate and accurate assessment of disease severity at diagnosis or during their early evolution is critical for reducing the risk of death in patients with CAP. Although CAP severity scores have been widely received and used to diagnose CAP patients, too many indices are needed to measure it, which makes it impractical in an emergency. Consequently, its application is restricted. It is very necessary to seek a new and rapid diagnostic method and biomarker for reducing morbidity and mortality in hospitalized patients.

Because the balance between oxidants and antioxidants is sufficient to maintain normal physiological functions, the excess production of oxidants or exhaustion of antioxidants can break the balance of the lung (7, 8). The imbalance between oxidants and antioxidants evokes oxidative stress. Earlier studies have found that oxidative stress is associated with many pulmonary inflammatory diseases (9–12). Oxidative damage to lipids (lipid peroxidation) leads to the production of by-products such as 8-iso-PGF2α (8-iso-prostaglandin F2α, 8-isoprostane), malondialdehyde, lipoperoxides, lipid hydroperoxides, and hydroxy non-enal alkyne. Due to their unstable and short half-life, it is difficult to determine free radicals. 8-Iso-PGF2α, which is produced independently of the cyclooxygenase enzyme by the peroxidation of arachidonic acid, is catalyzed by free radicals (13). 8-Iso-PGF2α has been considered as the most reliable index to reflect oxidative stress *in vivo* because of its sensitivity and specificity (14).

An increasing number of studies have demonstrated that elevated concentrations of 8-iso-PGF2α is a common characteristic of pulmonary diseases and that it is involved in the progression of these diseases, such as chronic obstructive pulmonary disease (COPD) (15), interstitial lung disease (16), acute lung injury (17), lung cancer (18), and respiratory failure (19). However, the associations of serum 8-iso-PGF2α on admission with the severity and prognosis are unclear among CAP patients. Although no study indicated the role of 8-iso-PGF2α on CAP. We speculate that 8-iso-PGF2α takes part in the pathophysiology of CAP. However, there is no clinical research clarifying the function of 8-iso-PGF2α in CAP. Furthermore, the correlations between serum 8-iso-PGF2α with the severity and prognosis were obscure in CAP patients. The primary goal of this study was consequently, to investigate the associations between serum 8-iso-PGF2α with the severity and prognosis of CAP patients in a hospital-based retrospective cohort study.

## Materials and Methods

### Subjects

This was a hospital-based retrospective cohort study. All CAP patients were admitted between May 2018 to May 2020 to the Second Affiliated Hospital of Anhui Medical University in Hefei City, Anhui Province, China. The Second Affiliated Hospital is an authoritative and large-scale general tertiary care University hospital in Anhui Province. All inpatients were enrolled from the Department of Respiratory and Critical Care Medicine. This project was approved by the Ethics Committee of the Second Affiliated Hospital of Anhui Medical University.

**Figure 1:**
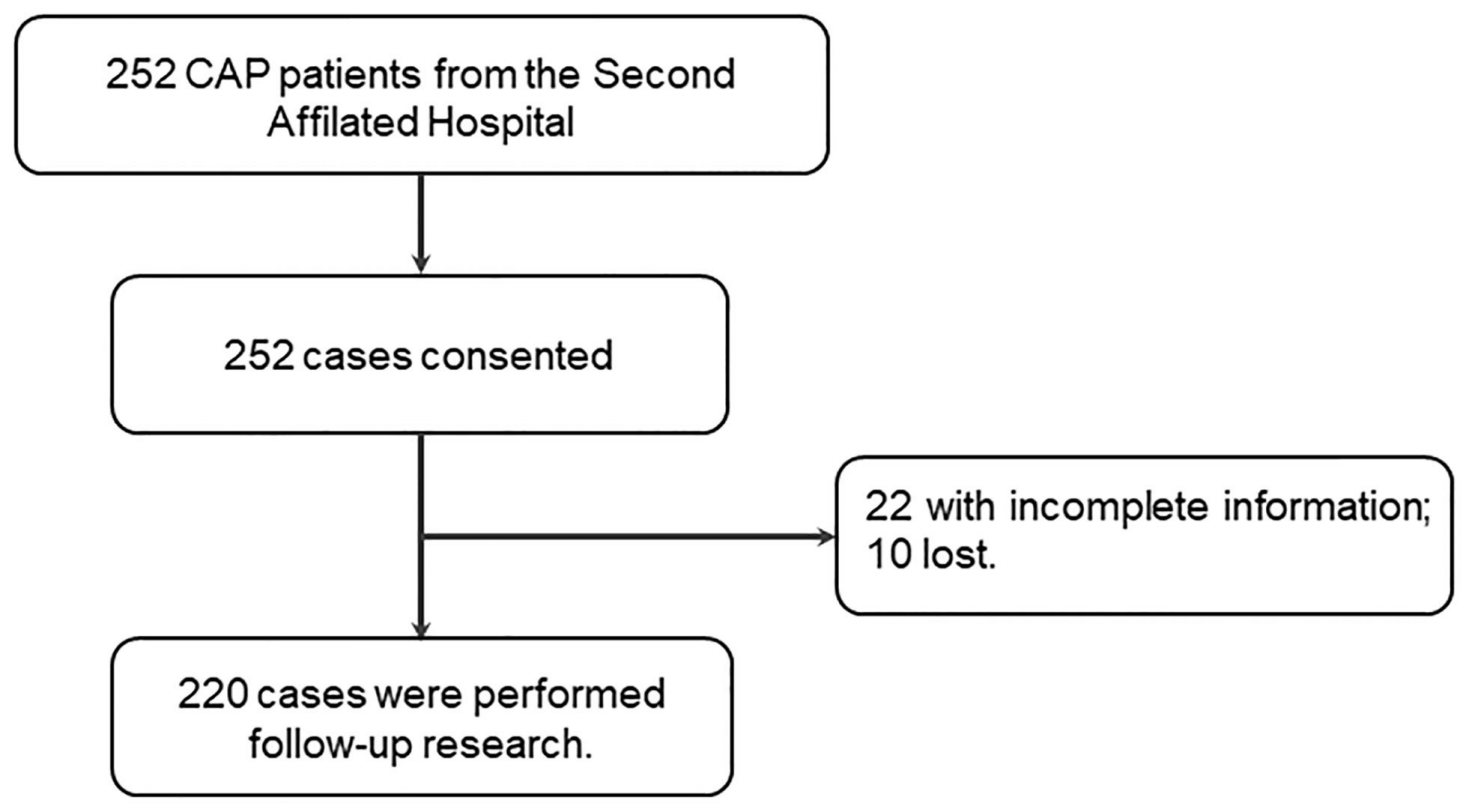
Flow diagram of recruitment and follow-up research in this cohort study.

Inclusion criteria were that participants should: be over 18 years old and have confirmed CAP for which they were subsequently hospitalized. Exclusion criteria included that they were an outpatient, had complications from other pulmonary diseases, such as malignancy, COPD, asthma, tuberculosis, were severely immunocompromised, had an organ or bone marrow transplant. All CAP patients were eligible for this research. we obtained written agreement consent from patients or the patient's next of kin. The patients with CAP were diagnosed in accordance with the presence of lower respiratory tract infection symptoms complicated with the appearance of obvious chest CT scan, and the absence of an alternative diagnosis. All 252 patients diagnosed with CAP were confirmed and recruited. Twenty-two patients with incomplete information and 10 patients who were lost to follow-up observation were excluded from this project. Finally, 220 patients with CAP participated in this research (Figure 1).

To evaluate the difference of serum 8-iso-PGF2α between Control subjects and CAP patients, 110 healthy volunteers, matched with age and age were recruited. Blood samples were collected among healthy subjects. On admission, blood samples of CAP patients were collected before treatment. Serum was centrifugated, sub-packed, and stored at −80°C in a super cold refrigerator (20). Demographic data and clinical information were acquired from medical records. The severity of pneumonia was evaluated by the CAP severity scores, including pneumonia severity index (PSI), CURB-65, CRB-65, CURXO, and SMART-COP (21).

### Enzyme-Linked Immunosorbent Assay

Specific commercial ELISA kits (8-iso-PGF2α) were purchased from Wuhan ColorfulGene Biological Technology Co., Ltd. Free 8-iso-PGF2α in serum were measured according to the manufacturer's protocol (22). Commercial ELISA kits (CRP, TNF-α, IL-1β, and IL-6) came from Cusabio, Wuhan, China (https://www.cusabio.com/) and were used to detect the levels of circulating inflammatory cytokines. All inflammatory cytokines were measured in accordance with the manufacturer's protocol (23).

### Statistical Analysis

All statistical analysis was conducted using the Statistical Program for the Social Sciences (SPSS) software, version 22.0. The categorical variables were expressed with frequencies and percentages. The quantitative variables were represented with the mean and standard error of the mean for normally distributed data, and median (interquartile range) for non-normally distributed data. Analysis of the relationship between qualitative variables was performed with the chi-square test or Fisher's exact test, and a comparison was conducted through *t* test or non-parametric test. The correlations between serum 8-iso-PGF2α and inflammatory cytokines were evaluated using Spearman correlation analysis. The correlations between serum 8-iso-PGF2α with CAP severity scores and hospital stay were accessed through univariate and multivariate logistic regression. Cox regression analysis and Kaplan Meier analysis were used to determine the correlations between serum 8-iso-PGF2α and the death in CAP patients. *P* < 0.05 was considered statistically significant.

## Results

### Demographic and Clinical Information

Two hundred and twenty patients (50.0% males) aged between 19 and 97 years (median 66.5 years) were recruited for this project. The median level of 8-iso-PGF2α was 30.46 pg/mL.

The demographic data and clinical information were collected and analyzed among CAP patients. As shown in Table 1, patients with CAP presenting higher and lower serum 8-iso-PGF2α levels (split at the median level) were similar in terms of gender, age, BMI, systolic pressure, and diastolic pressure. Moreover, routine blood parameters were analyzed in all patients with CAP. The numbers of white blood cells (WBC) and neutrophils, platelet-lymphocyte (PLR) ratio, neutrophil-lymphocyte (NLR) ratio, and monocyte-lymphocyte (MON) ratio were decreased, the numbers of lymphocytes were increased in patients with lower serum 8-iso-PGF2α than in patients with higher serum 8-iso-PGF2α (Table 1).

**Table 1:** Demographic and biochemical characteristics of CAP patients (split by median level of 8-iso-PGF2α).

**Variables**	**8-iso-PGF2α < median (110)**	**8-iso-PGF2α ≥ median (110)**	***P***
Male, *n* (%)	56 (51.0)	54 (49.0)	0.254
Age (years)	55.63 ± 2.77	71.55 ± 2.03	0.031
BMI	23.0 ± 0.52	22.4 ± 0.77	0.354
Systolic pressure (mmHg)	119.5 ± 2.39	125.9 ± 3.22	0.214
Diastolic pressure (mmHg)	74.5 ± 1.54	72.4 ± 1.42	0.198
WBC (10^9^/L)	7.67 ± 0.91	8.90 ± 0.63	0.006
Neutrophil (10^9^/L)	6.87 ± 1.61	7.03 ± 0.58	0.002
Lymphocyte (10^9^/L)	1.96 ± 0.43	1.13 ± 0.09	0.001
NLR	5.28 ± 1.13	8.07 ± 0.88	<0.001
MON	0.33 ± 0.03	0.57 ± 0.07	0.005
PLR	205.1 ± 23.06	257.6 ± 19.32	0.022
ALT (U/L)	31.90 ± 4.47	24.7 ± 3.05	0.042
AST (U/L)	32.33 ± 4.47	31.49 ± 3.11	0.568
Total bilirubin (umol/L)	13.31 ± 1.66	13.20 ± 1.08	0.554
Albumin (g/L)	35.43 ± 0.93	30.66 ± 0.83	0.852
Total bile acid (umol/L)	3.83 ± 0.64	4.53 ± 0.77	0.479
Urea nitrogen (mmol/L)	5.56 ± 0.35	6.49 ± 0.58	0.036
Creatinine (μmol/L)	61.22 ± 5.14	74.82 ± 7.92	0.035
Uric acid (μmol/L)	281.7 ± 12.30	276.7 ± 19.53	0.059
TNFα (pg/mL)	549.9 ± 56.30	930.3 ± 83.22	0.001
IL-1β (pg/mL)	359.2 ± 32.58	593.5 ± 79.33	0.013
IL-6 (pg/mL)	244.4 ± 17.59	164.8 ± 52.28	0.018
CRP (mg/L)	38.5 ± 8.51	77.9 ± 11.24	0.043
CURB-65	1.0 (0, 2.0)	2.0 (1.0, 4.0)	<0.001
CRB-65	1.0 (0, 1.75)	2.0 (1.0, 3.0)	<0.001
PSI	63.0 (45.0, 96.8)	123.0 (82.0, 151.0)	<0.001
CURXO [Severe, *n* (%)]	29 (26.4)	45 (40.1)	0.022
SMART-COP	1.0 (0, 2.0)	5.0 (1.0, 7.0)	<0.001

Additionally, liver function and renal function were assessed in all patients. Patients with lower 8-iso-PGF2α levels had lower levels of ALT, AST, urea nitrogen, and creatinine. There was no difference in total bilirubin, albumin, total bile acid, and uric acid levels observed between higher 8-iso-PGF2α and lower 8-iso-PGF2α patients with CAP (Table 1). In addition, serum inflammatory cytokines were determined. The levels of tumor necrosis factor (TNF)-α, interleukin (IL)-6, IL-1β, and C-reactive protein (CRP) were increased in patients with higher 8-iso-PGF2α levels (Table 1). Furthermore, disease severity was evaluated using CAP severity scores. As shown in Table 1, 45 (40.1%) cases were severe in CAP patients with higher 8-iso-PGF2α. Moreover, CAP patients with lower 8-iso-PGF2α had 29 (26.4%) severe cases. The CURB-65, CRB-65, CURXO, and SMART-COP scores were elevated in patients with higher 8-iso-PGF2α levels compared with patients with lower 8-iso-PGF2α levels.

### Serum 8-iso-PGF2α Levels in Patients With CAP and Control Subjects

**Figure 2:**
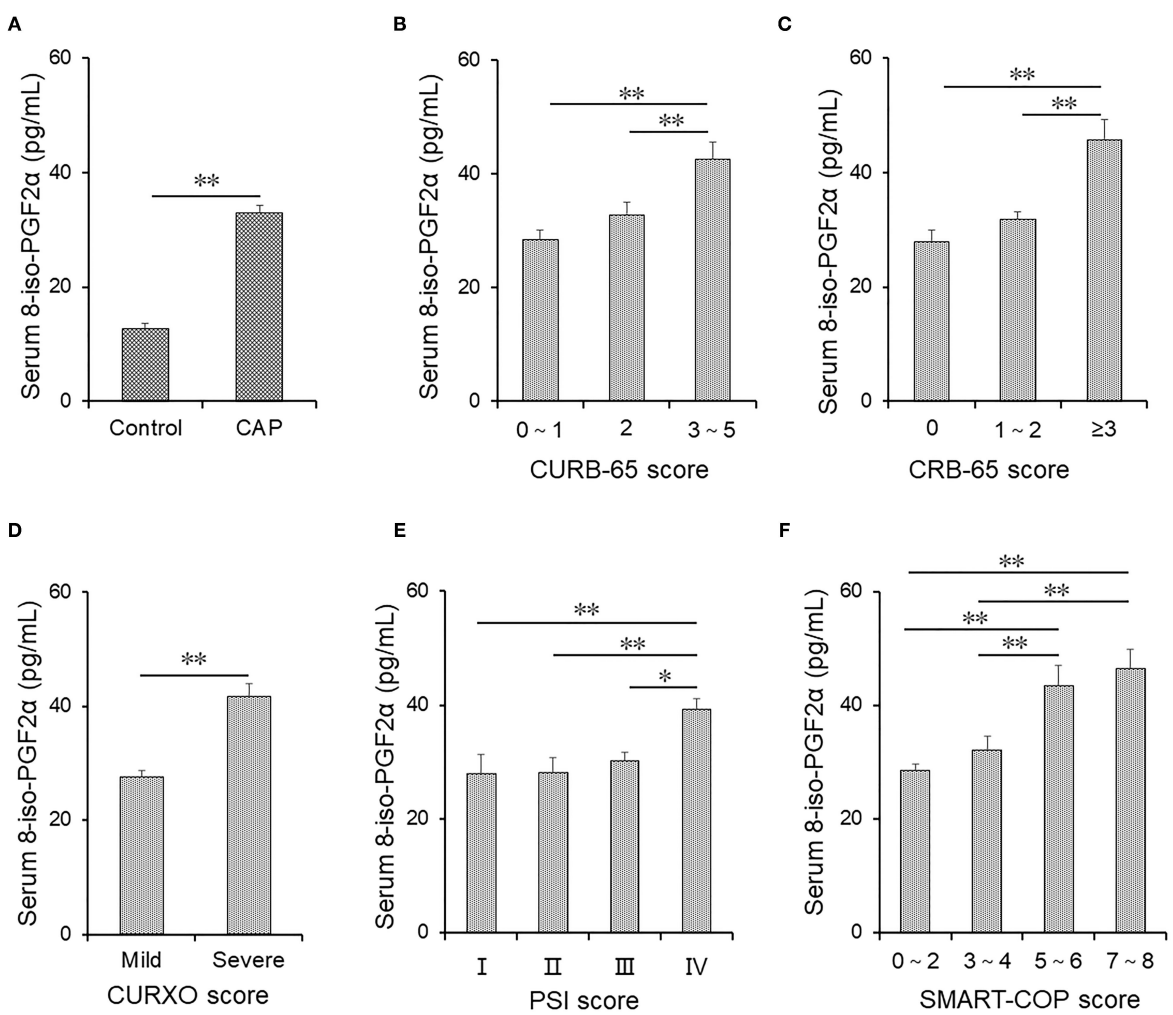
The levels of serum 8-iso-PGF2α in patients with CAP and Control subjects. **(A–F)** Serum 8-iso-PGF2α was measured using ELISA in CAP patients and Control subjects. **(A)** The levels of serum 8-iso-PGF2α were compared in patients with CAP and Control subjects. **(B)** The levels of serum 8-iso-PGF2α according to different grades of the CURB-65 score in patients with CAP. **(C)** The levels of serum 8-iso-PGF2α in different grades of CRB-65 score in patients with CAP. **(D)** The levels of serum 8-iso-PGF2α in different grades of the CURXO score in patients with CAP. **(E)** The levels of serum 8-iso-PGF2α in different grades of PSI score in patients with CAP. **(F)** The levels of serum 8-iso-PGF2α in different grades of SMART-COP score in patients with CAP. All data were expressed as mean ± SEM. *P < 0.05, **P < 0.01.

One hundred age- and sex-matched healthy volunteers were enrolled. Serum 8-iso-PGF2α levels were compared in CAP patients and Control subjects. As shown in Figure 2A, serum 8-iso-PGF2α was elevated in patients with CAP. Moreover, Serum 8-iso-PGF2α levels were detected in patients with different grades of CAP. As shown in Figure 2B, higher serum 8-iso-PGF2α levels were detected in patients with grade 3–5 CAP than in patients with grade 2 CAP, as defined according to the CURB-65 severity score.

Te serum 8-iso-PGF2α levels in patients with different grades of CAP defined according to the CRB-65 score were analyzed. The serum 8-iso-PGF2α levels were increased in patients with grade ≥3 CAP compared with patients with grades 0 and 1–2 CAP (Figure 2C). In addition, higher serum 8-iso-PGF2α levels were detected in patients with severe CAP than in patients with mild CAP (CURXO score) (Figure 2D). According to the PSI score, serum 8-iso-PGF2α levels were higher in patients with grade IV CAP than in patients with other grades of CAP (Figure 2E). The patients with CAP were divided into different grades based on the SMART-COP score. Higher serum 8-iso-PGF2α levels were detected in patients with grades 5–6 and 7–8 CAP than in patients with grades 1–2 and 3–4 CAP (Figure 2F).

### Associations of Serum 8-iso-PGF2α With Circulating Inflammatory Cytokines in Patients With CAP

The correlations between serum 8-iso-PGF2α and circulating inflammatory cytokines were explored using Spearman correlation analysis among CAP patients. As shown in Table 2, we found that serum 8-iso-PGF2α was positively correlated with CRP (*r* = 0.330, *P* = 0.003), TNF-α (*r* = 0.389, *P* = 0.001), and IL-6 (*r* = 0.299, *P* = 0.001) in CAP patients. There was no association between serum 8-iso-PGF2α and IL-6 among CAP patients.

**Table 2:** Associations between serum 8-iso-PGF2α and inflammatory cytokines among CAP patients.

**Variables**	***r***	***P***
CRP	0.330	0.003
TNF-α	0.389	0.001
IL-1β	0.299	0.001
IL-6	−0.043	0.764

### Association of Serum 8-iso-PGF2α Levels With Routine Blood Parameters in Patients With CAP

The correlations between serum 8-iso-PGF2α levels and routine blood parameters were determined using Spearman correlation analysis among CAP patients. As shown in Table 3, serum 8-iso-PGF2α was positively correlated with WBC (*r* = 0.229, *P* = 0.024) and neutrophil (*r* = 0.289, *P* = 0.004). Moreover, we found that serum 8-iso-PGF2α was inversely correlated with lymphocytes (*r* = −0.335, *P* = 0.001). There were also positive correlations between serum 8-iso-PGF2α and NLR (*r* = 0.354, *P* < 0.001), MON (*r* = 0.210, *P* = 0.038), and PLR (*r* = 0.244, *P* = 0.015).

**Table 3:** Associations between serum 8-iso-PGF2α and blood routine parameters among CAP patients.

**Variables**	***r***	***P***
WBC	0.229	0.024
Neutrophil	0.289	0.004
Lymphocyte	−0.335	0.001
NLR	0.354	<0.001
MON	0.210	0.038
PLR	0.244	0.015

### Association of Serum 8-iso-PGF2α Levels With CAP Severity Scores in Patients With CAP

The correlations between serum 8-iso-PGF2α levels and CAP severity scores among patients with CAP were determined using univariate and multivariate logistic regression analyses. As shown in Table 4, the univariate logistic regression analysis indicated that serum 8-iso-PGF2α levels were significantly and positively correlated with CURB-65 (OR: 1.095; 95% CI: 1.032–1.221), CRB-65 (OR: 1.308; 95% CI: 1.121–1.564), PSI (OR: 1.189; 95% CI: 1.012–1.356), SMART-COP (OR: 1.156; 95% CI: 1.085–1.305) and CURXO (OR: 1.189; 95% CI: 1.112–1.359).

**Table 4:** Associations between serum 8-iso-PGF2α with CAP severity scores among CAP patients.

**Variables**	**Univariate (95% *CI*)**	***P***	Multivariate (95% *CI*)*	***P***
CURB-65	1.095 (1.032, 1.221)	0.005	1.135 (1.056, 1.198)	0.038
CRB-65	1.308 (1.121, 1.564)	<0.001	1.312 (1.065, 1.535)	<0.001
PSI	1.189 (1.012, 1.356)	0.002	1.212 (1.019, 1.432)	0.009
SMART-COP	1.156 (1.085, 1.305)	0.021	1.165 (0.895, 1.912)	0.201
CURXO	1.189 (1.112, 1.359)	<0.001	1.156 (1.049, 1.237)	0.021

* *Adjusted for age, sex, neutrophil and lymphocyte*.

Multivariate logistic regression was conducted after adjustment for potential confounders. The level of serum 8-iso-PGF2α was significantly and positively associated with the CURB-65 (OR: 1.135; 95% CI: 1.056–1.198), CRB-65 (OR: 1.312; 95% CI: 1.065–1.535), PSI (OR: 1.212; 95% CI: 1.019–1.432), and CURXO (OR: 1.156; 95% CI: 1.049–1.237). No association was identified between serum 8-iso-PGF2α level and SMART-COP score (Table 4).

### Higher 8-iso-PGF2α Levels Prolong Hospital Stay and Increase the Risk of Death in Patients With CAP

**Figure 3:**
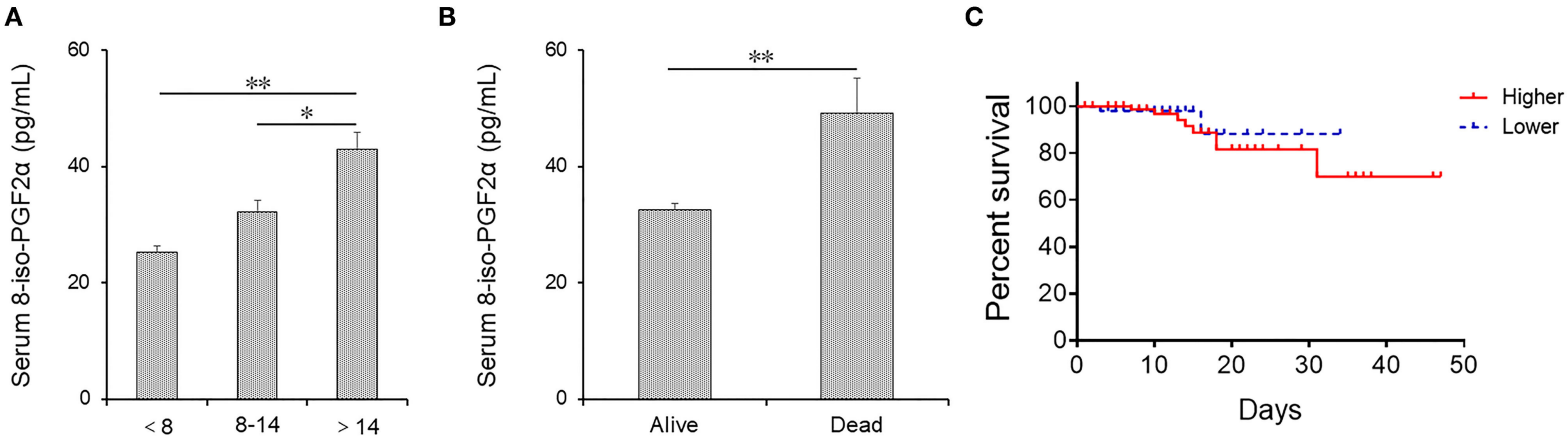
The levels of serum 8-iso-PGF2α in alive and dead CAP patients. **(A,B)** Serum 8-iso-PGF2α was measured using ELISA. **(A)** The levels of serum 8-iso-PGF2α in CAP patients with different lengths of hospital stay. **(B)** The levels of serum 8-iso-PGF2α in alive and dead patients. **(C)** Kaplan Meier plot of the probability survival of CAP patients during hospitalization. All data were expressed as mean ± SEM. *P < 0.05, **P < 0.01.

Serum 8-iso-PGF2α levels were measured in CAP patients with different hospital stays. As shown in Figure 3A, serum 8-iso-PGF2α levels were increased in patients whose hospital stay was more than 14 days than those whose hospital stay was <8 days and between 8 and 14 days. Moreover, 20 patients died from CAP after hospitalization. Serum 8-iso-PGF2α levels were compared between survival cases and dead patients. Serum 8-iso-PGF2α levels were reduced in survival patients compared with dead patients (Figure 3B).

The effects of serum 8-iso-PGF2α levels on death risk were analyzed among CAP patients. As shown in Table 5, Kaplan Meier analysis revealed that serum 8-iso-PGF2α on admission displayed a markedly positive association with the risk of death (HR: 1.122; 95% CI: 1.018–1.354). Additionally, after adjusted age and sex, Cox regression analysis indicated that higher 8-iso-PGF2α levels on admission were positively associated with the risk of death (HR: 1.189; 95% CI: 1.035–1.1366). The Kaplan Meier plot suggested that CAP patients with higher 8-iso-PGF2α levels had a higher mortality rate than those with lower 8-iso-PGF2α levels on admission (Figure 3C).

**Table 5:** Associations between serum 8-iso-PGF2α with prognosis among CAP patients.

**Variables**	**Kaplan Meier (95% CI)**	***P***	Cox regression (95% CI)*	***P***
Death	1.122 (1.018, 1.354)	0.011	1.189 (1.035, 1.366)	0.045

* *Adjusted for age and sex*.

## Discussion

To our knowledge, this is the first epidemiological study to explore the correlation between serum 8-iso-PGF2α and the severity and prognosis in patients with CAP. The major findings of this research are: (1) Serum 8-iso-PGF2α levels were higher in the severer patients with CAP. (2) Serum 8-iso-PGF2α levels were positively associated with circulating inflammatory cytokines in CAP patients. (3) Serum 8-iso-PGF2α levels were positively associated with CAP severity scores. (4) Higher serum 8-iso-PGF2α levels on admission prolonged the hospital stay and increased the risk of death in patients with CAP.

8-Iso-PGF2α is the product of an imbalance between oxidants and antioxidants in the body. The levels of 8-iso-PGF2α always reflect the status of oxidative stress *in vivo* (14). According to previous studies, 8-iso-PGF2α plays important roles in several respiratory diseases, and the levels of 8-iso-PGF2α are increased in patients with COPD (15), interstitial lung disease (16), acute lung injury (17), lung cancer (18), and respiratory failure (19). However, the correlations between serum 8-iso-PGF2α levels with the severity and prognosis remain obscure in CAP patients. In the present study, we found that serum 8-iso-PGF2α levels were increased in parallel with CAP severity scores among CAP patients. Further univariate and multivariate logistic regression analyses suggested that serum 8-iso-PGF2α levels were obviously and positively associated with disease severity in patients with CAP. These results indicate that 8-iso-PGF2α may be involved in the pathophysiology of CAP.

An increasing number of studies have revealed that pneumonia-induced pulmonary hyperpermeability is attributed to both the direct effects of pathogenic factors and uncontrolled host response. Some blood routine parameters and the ratios of different cell types (NLR, MON, and PLR) have been widely used to predict the prognosis of patients with CAP in recent studies (24, 25). Lymphocytes play an important role in fighting infection and motoring abnormal cells in the body. In terms of host factors, neutrophil recruitment increases the release of proinflammatory cytokines, which may contribute to the pathogenesis of CAP (25, 26). Uncontrolled excessive inflammation, neutrophil activation, and/or lymphopenia may lead to inflammatory cytokine storms and subsequent lung injury (27, 28).

Lymphopenia and elevated neutrophil counts were reported to be associated with a poor prognosis of patients with CAP (29). The lymphocyte count was reduced and neutrophil count was increased in patients with CAP with higher serum 8-iso-PGF2α levels. Additionally, NLR, MON, and PLR were elevated in patients with CAP presenting higher serum 8-iso-PGF2α. Further association analyses found that serum 8-iso-PGF2α levels were positively associated with neutrophil counts and negatively associated with lymphocyte counts. Moreover, accumulating data has clarified that circulating inflammatory cytokines are increased in patients with CAP (30, 31). In the present study, the levels of inflammatory cytokines were substantially increased in patients with CAP, presenting with higher serum 8-iso-PGF2α levels than those with lower 8-iso-PGF2α. In addition, logistic regression analysis discovered that serum 8-iso-PGF2α levels were notably and positively correlated with circulating inflammatory cytokines before and after adjustment for confounders. Moreover, elevating serum 8-iso-PGF2α levels on admission was positively associated with hospital stay and the risk of death in patients with CAP. These results provide evidence that serum 8-iso-PGF2α levels may represent a potential prognostic biomarker for patients with CAP.

8-Iso-PGF2α is a prostaglandin-like compound that is synthesized *in vivo*, mainly through a free radical–catalyzed peroxidation of arachidonic acid in cell membrane phospholipids. 8-Iso-PGF2α is then released to free form in serum, secretions, and urine by the action of phospholipase A2 (32). 8-Iso-PGF2α is a chemically stable end product of lipid peroxidation.

Based on accumulating evidence, oxidative stress may be involved in the pathobiology of CAP (14). Oxidative stress exerts an important role in the innate immune response, enabling the host to defend against external pathogens and evoking the secretion of inflammation mediators in the respiratory system (33). Oxidative stress also induces the production of cellular reactive oxygen species (ROS), causing cell, tissue damage, and then inflammation (34, 35). 8-Isoprostane has been widely viewed as the most reliable biomarker of lipid peroxidation due to ROS. A previous study indicated that ROS was increased in CAP patients (36, 37). ROS can peroxidize polyunsaturated fatty acids (PUFAs) to finally generate malondialdehyde and 4-hydroxy-2-nonenal. The eicosanoid PUFA arachidonic acid is peroxidized by ROS to form 8-isoprostane. An animal experiment revealed that 8-iso-PGF2α and inflammatory cytokines were increased in the chlorine inhalation-induced acute lung injury of mice (17). Urinary 8-iso-PGF2α were positively correlated with inflammatory cytokine levels in obese children (38). A randomized controlled trial indicated that the administration of N-acetylcysteine reduced oxidative and inflammatory damage in patients with pneumonia (39). Taking these studies into account, we speculate that 8-iso-PGF2α may play a detrimental role in the process of CAP. Levels of 8-iso-PGF2α could be used to reflect the levels of oxidative stress and inflammation in CAP patients.

There were limitations to the present study. (1) The number of participants was from a single center and relatively small. Larger sample sizes from multiple centers are required in future studies. Moreover, CAP patients who were not in hospital were not investigated, and other studies should resolve this by including outpatients in research. (2) The level of 8-iso-PGF2α was only detected in the serum of CAP patients. However, the levels of 8-iso-PGF2α in the sputum, bronchoalveolar lavage fluid, urine, and lung tissue of patients with CAP are unknown. Therefore, local levels of 8-iso-PGF2α will be measured in patients with CAP in future studies. (3) This study was only an epidemiological study and the causal relationship between 8-iso-PGF2α and inflammatory cytokine was unclear. Therefore, more laboratory studies are needed in the future. (4) Only free 8-iso-PGF2α was detected in the serum in this study but the total 8-iso-PGF2α in serum was unclear. (5) The level of serum 8-iso-PGF2α in CAP patients was only detected on admission. No follow-up measurement of 8-iso-PGF2α and no follow-up measurement of resulting data were observed among CAP patients.

## Conclusions

In conclusion, serum 8-iso-PGF2α levels on admission to hospital are positively associated with the disease severity and circulating inflammatory cytokines in patients with CAP. Higher serum 8-iso-PGF2α levels on admission prolong the hospital stay and increase the risk of death in patients with CAP. Based on these results, 8-iso-PGF2α may have an important role in the pathophysiology of CAP. Therefore, serum 8-iso-PGF2α levels may represent a prognostic biomarker and potential therapeutic target for CAP in clinical practice.

## Data Availability Statement

The raw data supporting the conclusions of this article will be made available by the authors, without undue reservation.

## Ethics Statement

Written informed consent was obtained from the individual(s) for the publication of any potentially identifiable images or data included in this article.

## Author Contributions

HZ and LF contributed to the design of the study, statistical analyses, and drafting the manuscript. LZ and JF contributed to sample collection, data interpretation and helped with drafting the manuscript. ZX and C-MF recruited patients and obtained their written informed consent. All authors participated in reviewing the manuscript and revising it critically before submission and read carefully and approved the final manuscript.

## Conflict of Interest

The authors declare that the research was conducted in the absence of any commercial or financial relationships that could be construed as a potential conflict of interest.

## Abbreviations

CAP: community-acquired pneumonia
8-iso-PGF2α: 8-iso-prostaglandin F2α
WBC: white blood cell
NLR: neutrophil-lymphocyte ratio
MON: monocyte-lymphocyte ratio
PLR: platelet-lymphocyte ratio
TNFα: tumor necrosis factor α
IL-1β: interleukin-1β
CRP: C-reactive protein.
